# Trends in colorectal cancer burden attributable to lifestyle in China (1990–2021): based on the global burden of disease study, revealing declining impact of dietary factors and rising influence of tobacco, alcohol, and obesity

**DOI:** 10.1186/s41043-026-01239-4

**Published:** 2026-01-21

**Authors:** Zhaofu Qin, Ziyan Weng, Ting Ma, Wenjun Li, Xinyi Gao, Dening Ma

**Affiliations:** 1 Department of Anesthesiology, The First Affiliated Hospital, Zhejiang University School of Medicine, Hangzhou, China; 2 Postgraduate training base Alliance of Wenzhou Medical University (Zhejiang Cancer Hospital), Hangzhou, 310022 China; 3 Department of Colorectal Surgery, Zhejiang Cancer Hospital, Hangzhou, 310022 China; 4 Department of Radiology, Zhejiang Cancer Hospital, Hangzhou, 310022 China

**Keywords:** Colorectal cancer, Global burden of disease, Lifestyle, China, Dietary pattern.

## Abstract

**Background:**

This study aimed to analyze trends in colorectal cancer (CRC) burden attributable to lifestyle factors in China (1990–2021), focusing on shifts of lifestyle, and to project future trajectories to inform public health strategies.

**Methods:**

Data from the Global Burden of Disease Study 2021 were utilized to assess deaths, disability-adjusted life years (DALYs), and age-standardized rates (ASRs) for CRC linked to nine lifestyle factors (including: diet low in whole grains, diet low in milk, diet low in fiber, diet low in calcium, diet high in red meat, diet high in processed meat, smoking, high alcohol use, and high BMI). Statistical analyses included estimated annual percentage change (EAPC), Joinpoint regression, age-period-cohort modeling, and Autoregressive Integrated Moving Average (ARIMA) projections (2022–2050).

**Results:**

Between 1990 and 2021, the burden of CRC attributable to most dietary factors declined, with significant reductions in low fiber (DALYs EAPC: -3.77) and low calcium intake (DALYs EAPC: -3.18). In contrast, processed meat intake showed an increase (DALYs EAPC: 1.64). Alcohol-related CRC burden rose slightly (DALYs EAPC: 0.35), while high BMI showed a marked increase (DALYs EAPC: 2.31). ARIMA projections suggest continued declines in dietary risk-related CRC burden. In contrast, the burden attributable to high body-mass index (BMI) is projected to rise substantially through 2050.

**Conclusions:**

While improved dietary habits have reduced CRC burden in China, rising obesity pose growing threats. Public health policies must prioritize interventions targeting processed meat intake, and weight management to curb future CRC incidence and mortality.

**Supplementary Information:**

The online version contains supplementary material available at 10.1186/s41043-026-01239-4.

## Contributions to the literature

While several studies have utilized Global Burden of Disease (GBD) data to examine risk factors for colorectal cancer (CRC) in China or globally, most have focused on single exposures (e.g., diet al.one or smoking alone) or provided static burden estimates without dynamic trend decomposition or future projections [1, 2]. In contrast, this study presents the first comprehensive analysis that simultaneously evaluate nine modifiable lifestyle factors—including dietary components (low whole grains, low milk, low fiber, low calcium, high red meat, high processed meat), smoking, alcohol use, and high BMI—in relation to CRC burden in China over a 32-year period (1990–2021). Moreover, we uniquely combine multiple advanced statistical approaches: estimated annual percentage change (EAPC) and Joinpoint regression for trend characterization, age-period-cohort (APC) modeling to disentangle temporal influences, and Autoregressive Integrated Moving Average (ARIMA) forecasting to project burden trajectories through 2050. This integrated framework enables a more nuanced understanding of shifting risk profiles and provides actionable evidence for prioritizing public health interventions.

## Background

Colorectal cancer (CRC) ranks as the third most common cancer globally, representing 10% of new cancer cases [3], and is the second leading cause of cancer-related deaths, accounting for 9.4% [4]. In China, the current landscape of CRC prevention and control remains significant challenges [5]. Despite advancements in comprehensive treatment modalities globally, primarily involving surgery, chemotherapy, and radiotherapy, which have improved the prognosis of CRC patients, both the incidence and mortality rates of CRC continue to rise [6]. Therefore, prevention remains the foremost strategy for mitigating the incidence and mortality associated with CRC.

Current studies indicate that the primary prevention, which involves addressing etiological factors and reducing exposure to risk factors through health education and the promotion of healthy lifestyles, is effective in alleviating the disease burden of CRC [7–9]. Lifestyle plays a critical role in the primary prevention of CRC, with established risk factors including dietary habits, tobacco and alcohol use, and weight management [10–14]. Empirical studies have identified several dietary risk factors associated with CRC, such as diets low in fiber and low calcium, as well as those high in red and processed meats. In recent years, insufficient consumption of milk and whole grains has emerged as a notable risk factor [15–17]. With the swift advancement of China’s economy and the gradual improvement of health awareness, it is hypothesized that the dietary patterns and lifestyle choices of the Chinese population are changing towards a healthier option. Consequently, it is anticipated that the burden of colorectal cancer, a disease closely related with these factors, will exhibit a corresponding decline.

This study aims to investigate the trend of colorectal cancer attributable to lifestyle risk factors through statistical analysis of data sourced from the Global Burden of Disease Study (GBD) database. The objective is to furnish robust evidence to inform the development of more precise and effective public health strategies aimed at reducing the incidence and mortality of CRC at its origin.

## Methods and materials

### Data collection

The Global Burden of Disease (GBD) 2021 represents an extensive international research collaboration dedicated to quantifying and comparing the global burden of various diseases, injuries, and risk factors. The GBD has provided data over a span of 32 years (1990–2021), encompassing a wide range of health outcome indicators, including deaths, disability-adjusted life years (DALYs), age-standardized rates (ASR), among others. Data were sourced from the Global Health Data Exchange website (https://ghdx.healthdata.org/gbd-2021), a publicly accessible data platform that enables researchers to access and analyze GBD data. This study specifically examined nine lifestyle risk factors associated with CRC, including: diet low in whole grains, diet low in milk, diet low in fiber, diet low in calcium, diet high in red meat, diet high in processed meat, smoking, high alcohol use, and high BMI. Measures such as DALYs, deaths, age-standardized mortality rates (ASMR) per 100,000 population and age-standardized DALYs rates (ASDR) per 100,000 population were used to evaluate the burden of CRC attributable to lifestyle factors in China. The data were categorized by age, forming subgroups at intervals of 5-year intervals, although the number of subgroups may vary depending on the specific risk variables. Since the GBD database utilizes publicly available data and does not involve the collection of personal information, ethical approval was not required.

### Definitions

In the Global Burden of Disease Study, CRC is defined as malignant neoplasms of the colon and rectum (ICD-10 codes C18–C21). Diet high in processed meat is defined as any intake (in grams per day) of meat preserved by smoking, curing, salting, or addition of chemical preservatives. Diet high in red meat is defined as intake above an average of 0 g per day (95% UI 0–200) of unprocessed red meat. The threshold of ‘>0 g/day’ for unprocessed red meat corresponds to the GBD 2021 theoretical minimum risk exposure level (TMREL), i.e., the intake at which the lowest CRC risk is assumed. Because any consumption above zero is considered avoidable, GBD assigns 100% of exposure above this level as attributable burden. Unprocessed red meat includes pork and bovine meats such as beef, lamb, and goat, but excludes all processed meats, poultry, fish, and eggs. Calcium intake is defined as average daily consumption of dietary calcium in grams per day from all sources, including milk, yoghurt, and cheese. The optimal intake for females is defined as 1.1–1.2 g per day, while the optimal intake for males is defined as 0.72–0.86 g per day. Diet low in fiber is defined as average daily consumption (in grams per day) of less than 22–25 g of fiber from all sources including fruits, vegetables, grains, legumes, and pulses. Milk intake is defined as average daily consumption in grams per day from all dairy milk sources, including non-fat, low‐fat, and full‐fat, and excluding soy milk and other plant derivatives. The optimal intake for females is defined as 500–610 g per day, while the optimal intake for males is defined as 280–340 g per day. Diet low in whole grains is defined as average daily consumption (in grams per day) of less than 160–210 g of whole grains (bran, germ, and endosperm in their natural proportion) from breakfast cereals, bread, rice, pasta, biscuits, muffins, tortillas, pancakes, and other sources. Smoking is defined as current daily or occasional use of any smoked tobacco product. Alcohol consumption in excess of the region, age, sex, and year-specific TMREL. Current drinkers are defined as individuals consuming at least one alcoholic beverage in the past year. We estimate the level of alcohol exposure for current drinkers with the reported average grams of pure alcohol consumed per day (g/day). High BMI for adults (ages 20 and older) is defined as BMI greater than 20–23 kg/m^2^. High BMI for children (ages 2–19) is defined as being overweight or obese based on International Obesity Task Force standards.

### Statistical analysis

The statistical analyses were conducted using R (version 4.2.1), Joinpoint (version 5.2.0), and Stata 16 software. Each statistical test was two-tailed, with a p-value threshold of less than 0.05 considered statistically significant. The estimated annual percentage change (EAPC) was employed to assess annual trends in ASDR per 100,000 population and ASMR per 100,000 population for CRC attributable to lifestyle habits in China between 1990 and 2021. To identify significant changes in the direction or magnitude of trends over the study period, we employed Joinpoint regression analysis. This model fits a series of connected linear segments to the time-series data, with the “Joinpoints” representing the points in time where a statistically significant change in the trend occurs. The optimal number of Joinpoints (i.e., inflection points) was determined using a permutation test, with a maximum of 6 joinpoints allowed. For each resulting segment, the model calculates an annual percentage change (APC), and an average annual percentage change (AAPC) is derived to summarize the trend over the entire 1990–2021 period. APC represents the annual rate of change in ASMR per 100,000 population and ASDR per 100,000 population over a specific period of time, and AAPC is an extension of APC to describe the average annual rate of change in the variables over the entire study period (1990–2021) [18].

Age-period-cohort analysis was used to analyze and explain the effects of three different time dimensions, age, time, and cohort, on the disease burden of CRC attributable to risk factors from lifestyles. The level of risk for a particular group of age, period, or cohort was measured by the Relative risk (*Exp* (coef) ). Age-period-cohort analysis was performed using the Intrinsic Estimator (IE) method in Stata 16 to address the identifiability problem inherent in APC models. To align with the standard 5-year interval grouping convention in APC analyses and ensure balanced period categories, we excluded data from 1990 to 1991, resulting in a 30-year analytical window (1992–2021) comprising six consecutive 5-year periods (1992–1996 through 2017–2021). This approach avoids interpolation of early-year data, which could introduce bias into birth cohort estimates.

In addition, the Autoregressive Integrated Moving Average (ARIMA) model was used to predict trends in DALY from 2022 to 2050 for colorectal cancer attributed to dietary risk, tobacco, alcohol, and high BMI, respectively. ARIMA model is a widely used model in time series analysis, which combines three parts: Autoregressive (AR), Integrated (I) and Moving Average (MA), and is suitable for non-stationary time series forecasting. For each lifestyle-attributable CRC burden indicator (e.g., DALYs due to high BMI, smoking, etc.), we identified the optimal ARIMA (p, d, q) model by minimizing the Akaike Information Criterion (AIC) and Bayesian Information Criterion (BIC). Final models were selected only if residuals passed the Ljung–Box test for white noise (*p* > 0.05). The specific (p, d, q) configurations for each risk factor are provided in S Table 14. It should be noted that the ARIMA model is a purely statistical time-series forecasting approach that relies solely on historical patterns in the data. As such, it does not account for potential future changes in public health policies, demographic shifts (e.g., aging populations), healthcare access, behavioral trends, or other exogenous factors that may substantially alter disease burden trajectories. Consequently, long-term projections—particularly those extending to 2050—should be interpreted as extrapolations of past trends under the assumption of structural stability, rather than definitive forecasts.

## Results

The number of deaths, DALYs, ASR related to CRC attributable to life behaviors in China from 1990 to 2021, and the EAPC representing their time trends are shown in Table 1. The EAPC results show that between 1990 and 2021, the burden of CRC in China attributable to the nine lifestyle factors exhibited divergent trends. Notably, three factors were associated with a statistically significant increase in CRC burden: high processed meat intake (DALYs EAPC: 1.64, 95% CI: 1.44, 1.83), high alcohol use (DALYs EAPC: 0.35, 95% CI: 0.05, 0.66), and high BMI (DALYs EAPC: 2.31, 95% CI: 2.19, 2.42). Conversely, significant decreasing trends were observed for low fiber intake (DALYs EAPC: -3.77, 95% CI: -3.86, -3.67) and low calcium intake (DALYs EAPC: -3.18, 95% CI: -3.28, -3.08). The burdens attributable to low whole grains, low milk, high red meat, and smoking showed slight declines or remained relatively stable.

**Table 1:** Analysis and Temporal trends of CRC deaths, DALYs, and ASRs associated with diet, tobacco, alcohol and weight, in China, 1990–2021

	1990		2021		EAPC (95% CI)
	Number	ASR per 100,000	Number	ASR per 100,000	
**Diet**					
***Diet low in whole grains***					
DALYs	624,948	69.54	1,241,928	59.7	-0.57 (-0.65 ,-0.5)
Deaths	21,330	2.79	49,991	2.47	-0.46 (-0.52 ,-0.41)
***Diet low in milk***					
DALYs	652,029	73.14	1,253,643	60.25	-0.73 (-0.82 ,-0.65)
Deaths	22,541	2.98	51,030	2.53	-0.63 (-0.7 ,-0.57)
***Diet high in red meat***					
DALYs	518,213	57.5	1,091,788	52.47	-0.33 (-0.41 ,-0.25)
Deaths	17,608	2.29	43,580	2.15	-0.24 (-0.3 ,-0.19)
***Diet high in processed meat***					
DALYs	53,168	5.83	178,187	8.57	1.64 (1.44 ,1.83)
Deaths	1765	0.23	6612	0.32	1.46 (1.31 ,1.6)
***Diet low in fiber***					
DALYs	68,081	7.32	48,100	2.4	-3.77 (-3.86 ,-3.67)
Deaths	2100	0.27	1739	0.09	-3.74 (-3.85 ,-3.62)
***Diet low in calcium***					
DALYs	546,369	61.59	500,468	24.2	-3.18 (-3.28 ,-3.08)
Deaths	18,903	2.55	20,719	1.04	-3.06 (-3.17 ,-2.96)
***Tobacco and alcohol***					
***Smoking***					
DALYs	232,204	25.32	459,250	21.44	-0.46 (-0.6 ,-0.31)
Deaths	7775	0.95	17,277	0.82	-0.37 (-0.48 ,-0.26)
***High alcohol use***					
DALYs	181,730	18.89	421,731	20.34	0.35 (0.05 ,0.66)
Deaths	5570	0.65	14,937	0.72	0.48 (0.2 ,0.75)
***Weight***					
***High BMI***					
DALYs	109,322	11.88	507,316	24.22	2.31 (2.19 ,2.42)
Deaths	3611	0.45	19,418	0.94	2.39 (2.31 ,2.48)

ASR: age-standardized rates; CRC: colorectal cancer; ASR: age-standardized rate; DALYs: disability-adjusted life years; EAPC: estimated annual percentage change; BMI: body-mass index

**Fig. 1:**
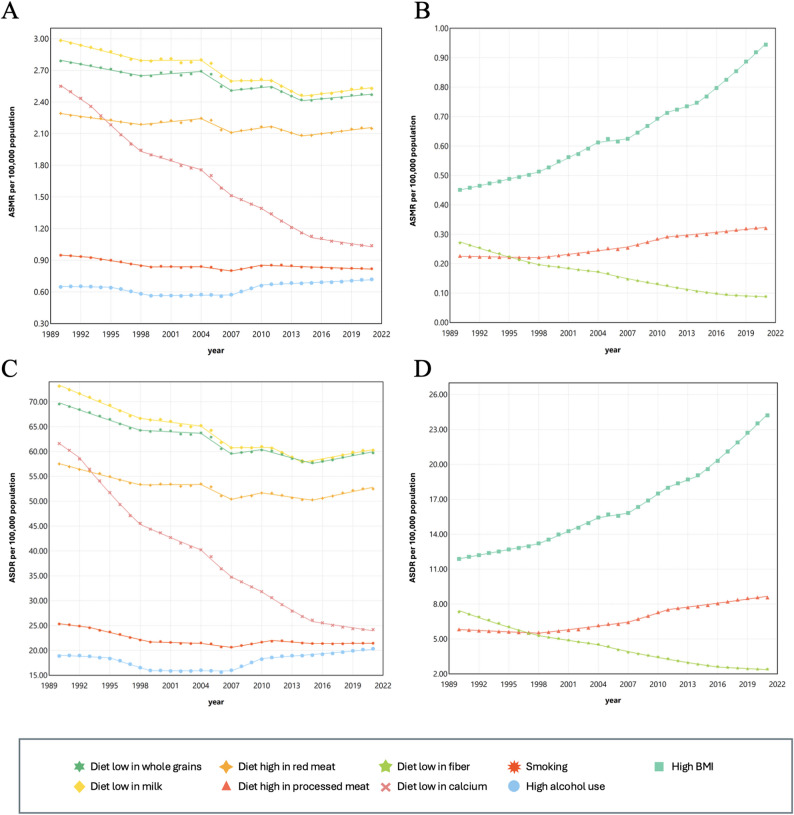
Temporal trends of CRC ASDR and ASMR attributed to diet, tobacco, alcohol, and weight in China by Joinpoint regression, 1990–2021. ASMR (**A**) and ASDR (**C**) based analysis of risk factors including diet low in whole grains, diet high in red meat, smoking, diet low in milk, diet low in calcium and high alcohol use. ASMR (**B**) and ASDR (**D**) based analysis of risk factors including diet low in fiber, diet high in processed meat and high BMI. ASDR : age-standardized DALYs rates; ASMR : age-standardized mortality rates. CRC: colorectal cancer; DALYs: disability-adjusted life years; BMI: body-mass index. Straight-line segments connected at statistically identified inflection years, indicating significant changes in trend direction or magnitude

The Joinpoint regression model analysis results of ASDR per 100,000 population and ASMR per 100,000 population for CRC attributable to lifestyles in China during 1990–2021 are shown in Fig. 1; Table 2, and Table 3. In general, the outcomes of ASMR per 100,000 population and ASDR per 100,000 population were comparable. The Joinpoint regression analysis yielded comparable findings for the risk factors of diet low in whole grains, diet low in milk and diet high in red meats, displaying a downward trend in general (diet low in whole grain: AAPC (ASMR per 100,000 population) = -0.39 (-0.42, -0.37), AAPC (ASDR per 100,000 population) = -0.49 (-0.52, -0.46); diet low in milk: AAPC (ASMR per 100,000 population) = -0.53 (-0.56, -0.5), AAPC (ASDR per 100,000 population) = -0.63 (-0.66, -0.6); diet high in red meat: AAPC (ASMR per 100,000 population) = -0.19 (-0.22, -0.16), AAPC (ASDR per 100,000 population) = -0.28 (-0.3, -0.25)), only showing an upward trend from 1998 to 2004 and from 2014 to 2021. What’s more, diet high in processed meat (AAPC (ASMR per 100,000 population) = 1.17 (1.13, 1.22), AAPC (ASDR per 100,000 population) = 1.30 (1.26, 1.33)) and high BMI (AAPC (ASMR per 100,000 population) = 2.43 (2.4, 2.45), AAPC(ASDR per 100,000 population) = 2.33 (2.31, 2.36)) showed an upward trend overall, but the difference was that in the early period (1990–1998), high-processed meat diet showed a declining trend, and then rose at varying rates in subsequent periods, while high BMI showed a more pronounced upward trend over every period. Furthermore, diet low in calcium (AAPC (ASMR per 100,000 population) = -2.90 (-2.96, -2.83), AAPC (ASDR per 100,000 population) = -3.00 (-3.05, -2.95)) and diet low in fiber (AAPC (ASMR per 100,000 population) = -3.61 (-3.7, -3.54), AAPC (ASDR per 100,000 population) = -3.63 (-3.71, -3.56)) demonstrated descending trends at different rates in varied periods. Trends in disease burden of smoking-induced CRC, while overall showing a decreasing trend, appear to have risen in recent years (AAPC (ASMR per 100,000 population) = -0.48 (-0.54, -0.43), AAPC (ASDR per 100,000 population) = -0.54 (-0.58, -0.5)). As for the risk factor for high alcohol use, while there was a declining trend in the early years, there has been a significant rise in recent years, leading to an overall upward trend (AAPC (ASMR per 100,000 population) = 0.30 (0.25, 0.35), AAPC (ASDR per 100,000 population) = 0.19 (0.14, 0.24)).

**Table 2:** Joinpoint regression analysis of CRC ASMR per 100,000 population attributable to diet, tobacco, alcohol and weight, in China, 1990–2021

		ASMR per 100,000 population							
		Segment 1	Segment 2	Segment 3	Segment 4	Segment 5	Segment 6	Segment 7	AAPC
**Diet**									
Diet low in whole grains	Period	1990–1998	1998–2004	2004–2007	2007–2011	2011–2014	2014–2021	/	1990–2021
	APC (95%)	-0.67* (-0.84 ,-0.55)	0.25* (0.07 ,0.58)	-2.28* (-2.6 ,-1.72)	0.36* (0.12 ,0.83)	-1.78* (-2.08 ,-1.23)	0.36* (0.22 ,0.54)	/	-0.39* (-0.42 ,-0.37)
	P-Value	*P* < 0.001	0.006	*P* < 0.001	0.007	*P* < 0.001	*P* < 0.001	/	*P* < 0.001
Diet low in milk	Period	1990–1998	1998–2004	2004–2007	2007–2011	2011–2014	2014–2021	/	1990–2021
	APC (95%)	-0.84* (-1.01 ,-0.71)	0.02 (-0.16 ,0.38)	-2.41* (-2.74 ,-1.86)	0.06 (-0.18 ,0.57)	-1.97* (-2.27 ,-1.39)	0.47* (0.33 ,0.63)	/	-0.53* (-0.56 ,-0.5)
	P-Value	*P* < 0.001	0.79	*P* < 0.001	0.53	*P* < 0.001	*P* < 0.001	/	*P* < 0.001
Diet high in red meat	Period	1990–1998	1998–2004	2004–2007	2007–2011	2011–2014	2014–2021	/	1990–2021
	APC (95%)	-0.59* (-0.77 ,-0.45)	0.43* (0.24 ,0.81)	-1.99* (-2.33 ,-1.39)	0.68* (0.42 ,1.23)	-1.43* (-1.74 ,-0.84)	0.55* (0.4 ,0.75)	/	-0.19* (-0.22 ,-0.16)
	P-Value	*P* < 0.001	*P* < 0.001	*P* < 0.001	*P* < 0.001	*P* < 0.001	*P* < 0.001	/	*P* < 0.001
Diet high in processed meat	Period	1990–1998	1998–2007	2007–2011	2011–2021	/	/	/	1990–2021
	APC (95%)	-0.28* (-0.54 ,-0.07)	1.71* (1.42 ,1.92)	3.17* (2.56 ,3.88)	1.07* (0.89 ,1.22)	/	/	/	1.17* (1.13 ,1.22)
	P-Value	*P* < 0.001	*P* < 0.001	*P* < 0.001	0.009	/	/	/	*P* < 0.001
Diet low in fiber	Period	1990–1998	1998–2004	2004–2017	2017–2021	/	/	/	1990–2021
	APC (95%)	-4.07* (-4.63 ,-3.76)	-2.24* (-2.76 ,-1.11)	-4.57* (-4.79 ,-4.41)	-1.59* (-2.54 ,-0.09)	/	/	/	-3.61* (-3.7 ,-3.54)
	P-Value	*P* < 0.001	*P* < 0.001	*P* < 0.001	0.043	/	/	/	*P* < 0.001
Diet low in calcium	Period	1990–1993	1993–1998	1998–2004	2004–2007	2007–2010	2010–2015	2015–2021	1990–2021
	APC (95%)	-2.60* (-3.46 ,-1.48)	-3.95* (-4.81 ,-1.77)	-1.56* (-4.21 ,-1.07)	-4.81* (-5.29 ,-2.84)	-2.78* (-4.31 ,-2.28)	-4.36* (-5.16 ,-2.19)	-1.34* (-1.78 ,-0.65)	-2.90* (-2.96 ,-2.83)
	P-Value	*P* < 0.001	*P* < 0.001	*P* < 0.001	*P* < 0.001	*P* < 0.001	0.002	0.006	*P* < 0.001
***Tobacco and alcohol***									
Smoking	Period	1990–1993	1993–1999	1999–2004	2004–2007	2007–2010	2010–2021	/	1990–2021
	APC (95%)	-0.76 (-1.45 ,0.23)	-1.69 (-2.59 ,0.17)	0.04 (-1.69 ,0.85)	-1.62 (-2.1 ,2.34)	2.22 (-0.68 ,2.64)	-0.40* (-0.6 ,-0.08)	/	-0.48* (-0.54 ,-0.43)
	P-Value	0.088	0.084	0.7	0.14	0.14	0.038	/	*P* < 0.001
High alcohol use	Period	1990–1995	1995–1999	1999–2007	2007–2010	2010–2021	/	/	1990–2021
	APC (95%)	-0.23 (-0.66 ,0.2)	-3.26* (-3.94 ,-2.76)	0.14 (-0.04 ,0.34)	5.27* (4.76 ,5.67)	0.65* (0.5 ,0.76)	/	/	0.30* (0.25 ,0.35)
	P-Value	0.24	*P* < 0.001	0.11	*P* < 0.001	*P* < 0.001	/	/	*P* < 0.001
***Weight***									
High BMI	Period	1990–1998	1998–2004	2004–2007	2007–2011	2011–2014	2014–2021	/	1990–2021
	APC (95%)	1.61* (1.47 ,1.73)	3.03* (2.85 ,3.28)	0.65* (0.37 ,1.12)	3.41* (3.2 ,3.79)	1.41* (1.15 ,1.92)	3.50* (3.38 ,3.65)	/	2.43* (2.4 ,2.45)
	P-Value	*P* < 0.001	*P* < 0.001	*P* < 0.001	*P* < 0.001	*P* < 0.001	*P* < 0.001	/	*P* < 0.001

**Table 3:** Joinpoint regression analysis of CRC ASDR per 100,000 population attributable to diet, tobacco, alcohol and weight, in China, 1990–2021

		ASDR per 100,000 population							
		Segment 1	Segment 2	Segment 3	Segment 4	Segment 5	Segment 6	Segment 7	AAPC
**Diet**									
Diet low in whole grains	Period	1990–1998	1998–2004	2004–2007	2007–2010	2010–2015	2015–2021	/	1990–2021
	APC (95%)	-1.03* (-1.19 ,-0.91)	-0.16 (-0.31 ,0.16)	-2.24* (-2.54 ,-1.76)	0.49* (0.07 ,0.8)	-0.94* (-1.37 ,-0.76)	0.67* (0.49 ,0.91)	/	-0.49* (-0.52 ,-0.46)
	P-Value	*P* < 0.001	0.178	*P* < 0.001	0.02	*P* < 0.001	*P* < 0.001	/	*P* < 0.001
Diet low in milk	Period	1990–1998	1998–2004	2004–2007	2007–2011	2011–2014	2014–2021	/	1990–2021
	APC (95%)	-1.19* (-1.42 ,-1.05)	-0.39 (-0.56 ,0.04)	-2.29* (-2.62 ,-1.78)	0.03 (-0.19 ,0.57)	-1.67* (-1.96 ,-1.16)	0.62* (0.47 ,0.81)	/	-0.63* (-0.66 ,-0.6)
	P-Value	*P* < 0.001	0.064	*P* < 0.001	0.72	0.001	*P* < 0.001	/	*P* < 0.001
Diet high in red meat	Period	1990–1998	1998–2004	2004–2007	2007–2010	2010–2015	2015–2021	/	1990–2021
	APC (95%)	-0.96* (-1.09 ,-0.85)	0.03 (-0.13 ,0.28)	-1.91* (-2.18 ,-1.48)	0.86* (0.46 ,1.14)	-0.58* (-0.94 ,-0.4)	0.85* (0.68 ,1.05)	/	-0.28* (-0.3 ,-0.25)
	P-Value	*P* < 0.001	0.674	*P* < 0.001	*P* < 0.001	*P* < 0.001	*P* < 0.001	/	*P* < 0.001
Diet high in processed meat	Period	1990–1998	1998–2007	2007–2011	2011–2021	/	/	/	1990–2021
	APC (95%)	-0.69* (-0.87 ,-0.52)	1.78* (1.59 ,1.94)	3.94* (3.35 ,4.63)	1.42* (1.28 ,1.55)	/	/	/	1.30* (1.26 ,1.33)
	P-Value	*P* < 0.001	*P* < 0.001	*P* < 0.001	*P* < 0.001	/	/	/	*P* < 0.001
Diet low in fiber	Period	1990–1998	1998–2004	2004–2016	2016–2021	/	/	/	1990–2021
	APC (95%)	-4.19* (-4.83 ,-3.88)	-2.64* (-3.14 ,-1.58)	-4.51* (-4.75 ,-4.33)	-1.78* (-2.45 ,-0.79)	/	/	/	-3.63* (-3.71 ,-3.56)
	P-Value	*P* < 0.001	*P* < 0.001	*P* < 0.001	0.015	/	/	/	*P* < 0.001
Diet low in calcium	Period	1990–1992	1992–1998	1998–2004	2004–2007	2007–2010	2010–2015	2015–2021	1990–2021
	APC (95%)	-2.35* (-3.34 ,-1.57)	-4.23* (-4.73 ,-4.05)	-1.98* (-2.23 ,-1.68)	-4.69* (-5.05 ,-3.89)	-2.97* (-3.63 ,-2.57)	-4.10* (-4.72 ,-3.66)	-1.23* (-1.53 ,-0.87)	-3.00* (-3.05 ,-2.95)
	P-Value	*P* < 0.001	*P* < 0.001	*P* < 0.001	*P* < 0.001	*P* < 0.001	*P* < 0.001	0.002	*P* < 0.001
***Tobacco and alcohol***									
Smoking	Period	1990–1993	1993–1999	1999–2004	2004–2007	2007–2011	2011–2015	2015–2021	1990–2021
	APC (95%)	-0.98* (-1.4 ,-0.27)	-2.08* (-2.56 ,-1.91)	-0.26 (-0.5 ,0.2)	-1.27* (-1.59 ,-0.77)	1.64* (1.19 ,2.05)	-0.72* (-1.13 ,-0.29)	0.05 (-0.17 ,0.61)	-0.54* (-0.58 ,-0.5)
	P-Value	0.027	*P* < 0.001	0.134	0.016	0.027	0.026	0.524	*P* < 0.001
High alcohol use	Period	1990–1995	1995–1999	1999–2007	2007–2010	2010–2021	/	/	1990–2021
	APC (95%)	-0.55* (-1.02 ,-0.11)	-3.69* (-4.29 ,-3.17)	-0.04 (-0.22 ,0.15)	4.97* (4.06 ,5.39)	0.87* (0.72 ,0.99)	/	/	0.19* (0.14 ,0.24)
	P-Value	0.025	*P* < 0.001	0.622	*P* < 0.001	*P* < 0.001	/	/	*P* < 0.001
***Weight***									
High BMI	Period	1990–1998	1998–2004	2004–2007	2007–2011	2011–2014	2014–2021	/	1990–2021
	APC (95%)	1.30* (1.18 ,1.41)	2.64* (2.5 ,2.86)	0.84* (0.59 ,1.25)	3.35* (3.16 ,3.68)	1.73* (1.51 ,2.16)	3.58* (3.48 ,3.71)	/	2.33* (2.31 ,2.36)
	P-Value	*P* < 0.001	*P* < 0.001	*P* < 0.001	*P* < 0.001	*P* < 0.001	*P* < 0.001	/	*P* < 0.001

**Fig. 2:**
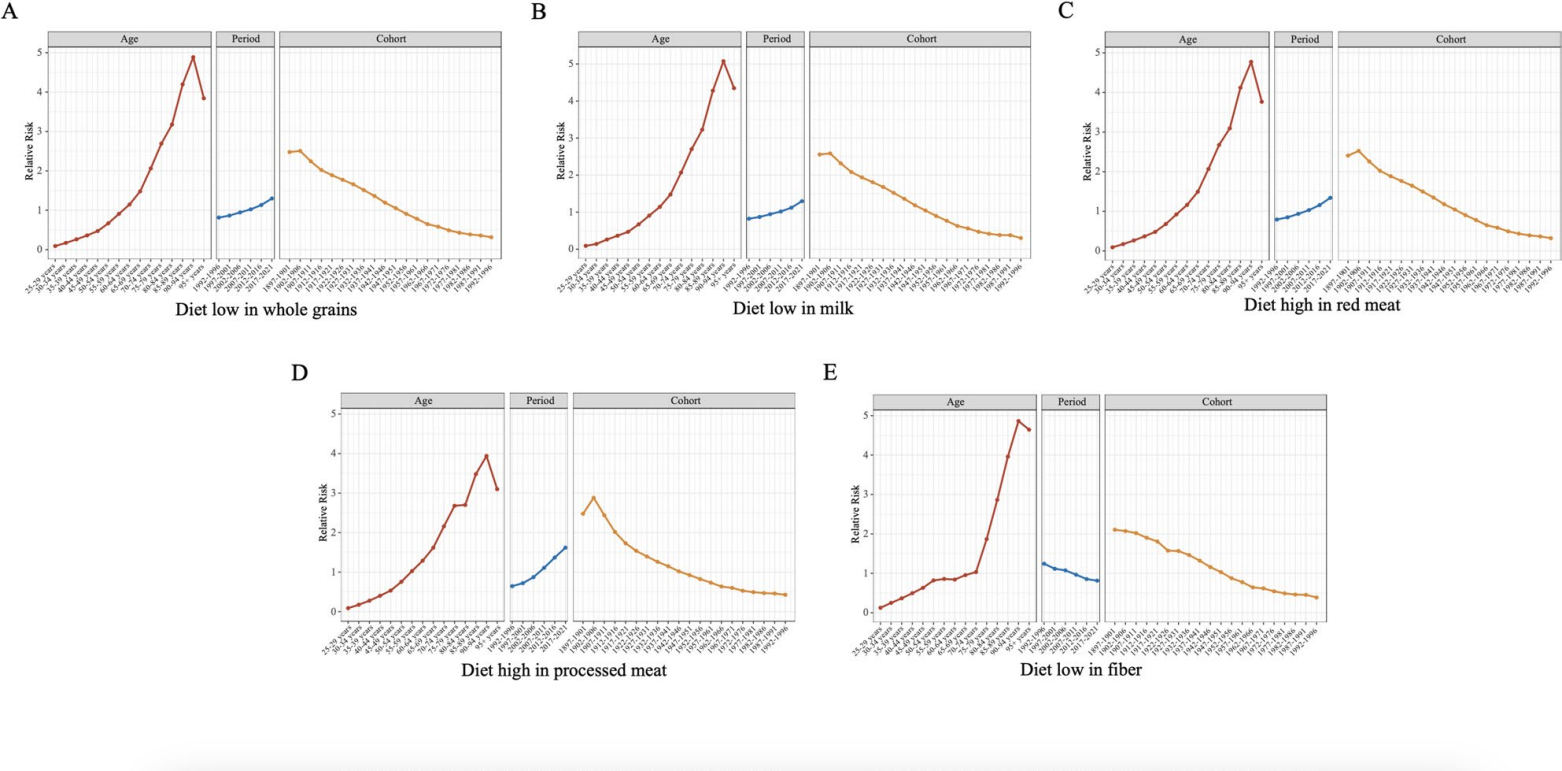
Age-period-cohort analysis of CRC attributed to diet low in whole grains (**A**), diet low in milk (**B**), diet high in red meat (**C**), diet high in processed meat (**D**) and diet low in fiber (E) based on ASMR in China, 1992–2021. The entries at the top of Figure exist to distinguish between age cohort, period cohort, and birth cohort. ASMR : age-standardized mortality rates. CRC: colorectal cancer; DALYs: disability-adjusted life years; BMI: body-mass index

**Fig. 3:**
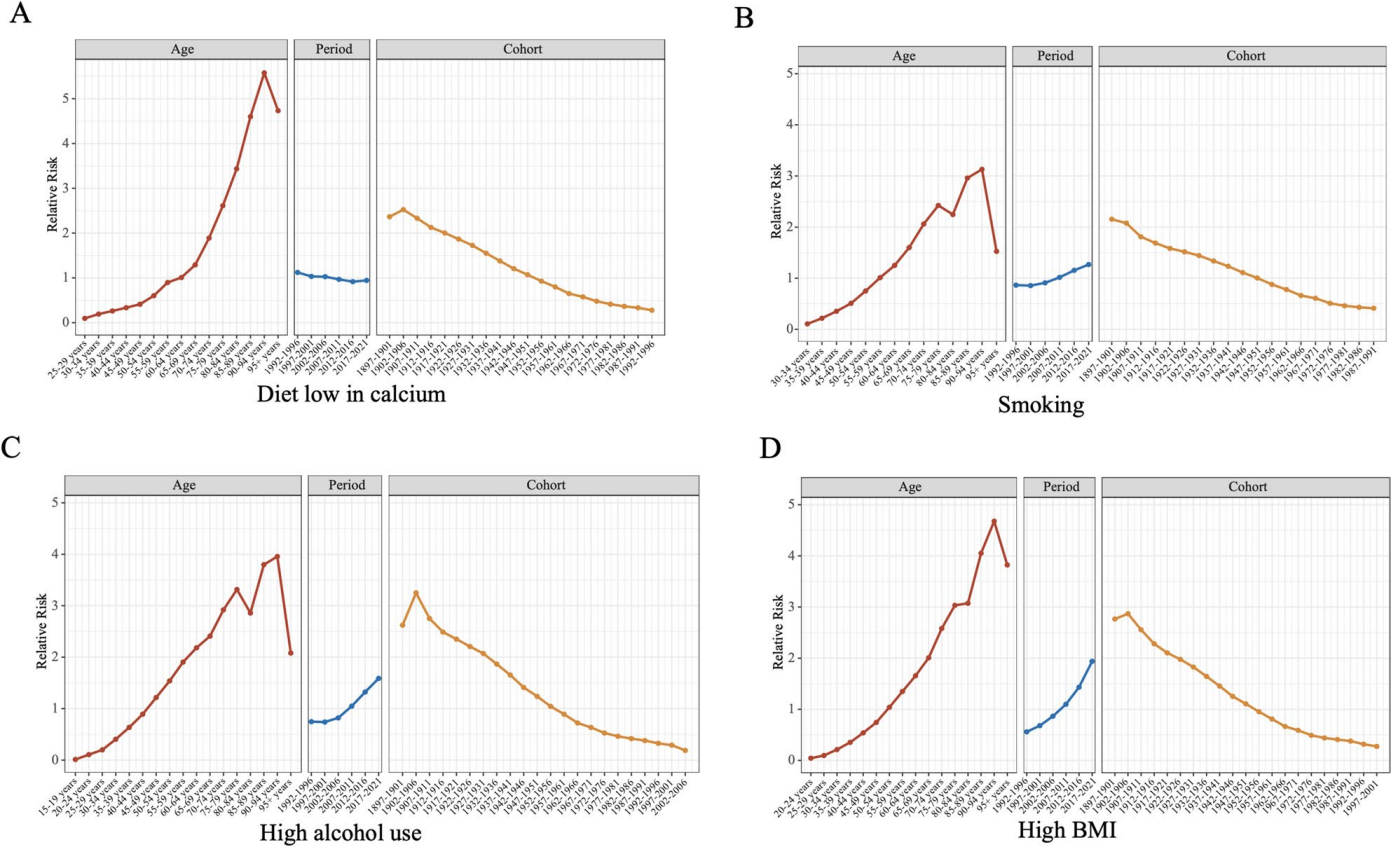
Age-period-cohort analysis of CRC attributed to diet low in calcium (**A**), smoking (**B**), high alcohol use (**C**) and high BMI (**D**) based on ASMR in China, 1992–2021. The entries at the top of Figure exist to distinguish between age cohort, period cohort, and birth cohort. ASMR : age-standardized mortality rates. CRC: colorectal cancer; DALYs: disability-adjusted life years; BMI: body-mass index

The relative risk calculated from the age-period-cohort analysis based on the ASMR per 100,000 population of CRC attributable to lifestyles in China during the period 1992–2021 is illustrated in Figs. 2 and 3 and Supplementary materials. The advanced age cohorts’ curves exhibited differing degrees of fluctuation in all analyses, which may be attributed to the low number of CRCs related to lifestyle habits in the elderly population. The same phenomenon can be observed in the earlier birth cohorts. In the analysis of age cohorts for all risk factors included in the study, we saw that the relative risk of CRC owing to lifestyle habits increased as age increased. Meanwhile, in the period cohort analyses, only the relative risks of diet low in fiber and diet low in calcium showed a negative correlation, which means that the risk of diet-attributable CRC is now lower than previously, and the relative risks of the period cohorts for the rest of the risk factors showed a markedly positive correlation. Finally, a key finding from the birth cohort analysis was that the relative risk (RR) of CRC showed a clear downward trend across successive birth cohorts for all nine lifestyle risk factors examined.

**Fig. 4:**
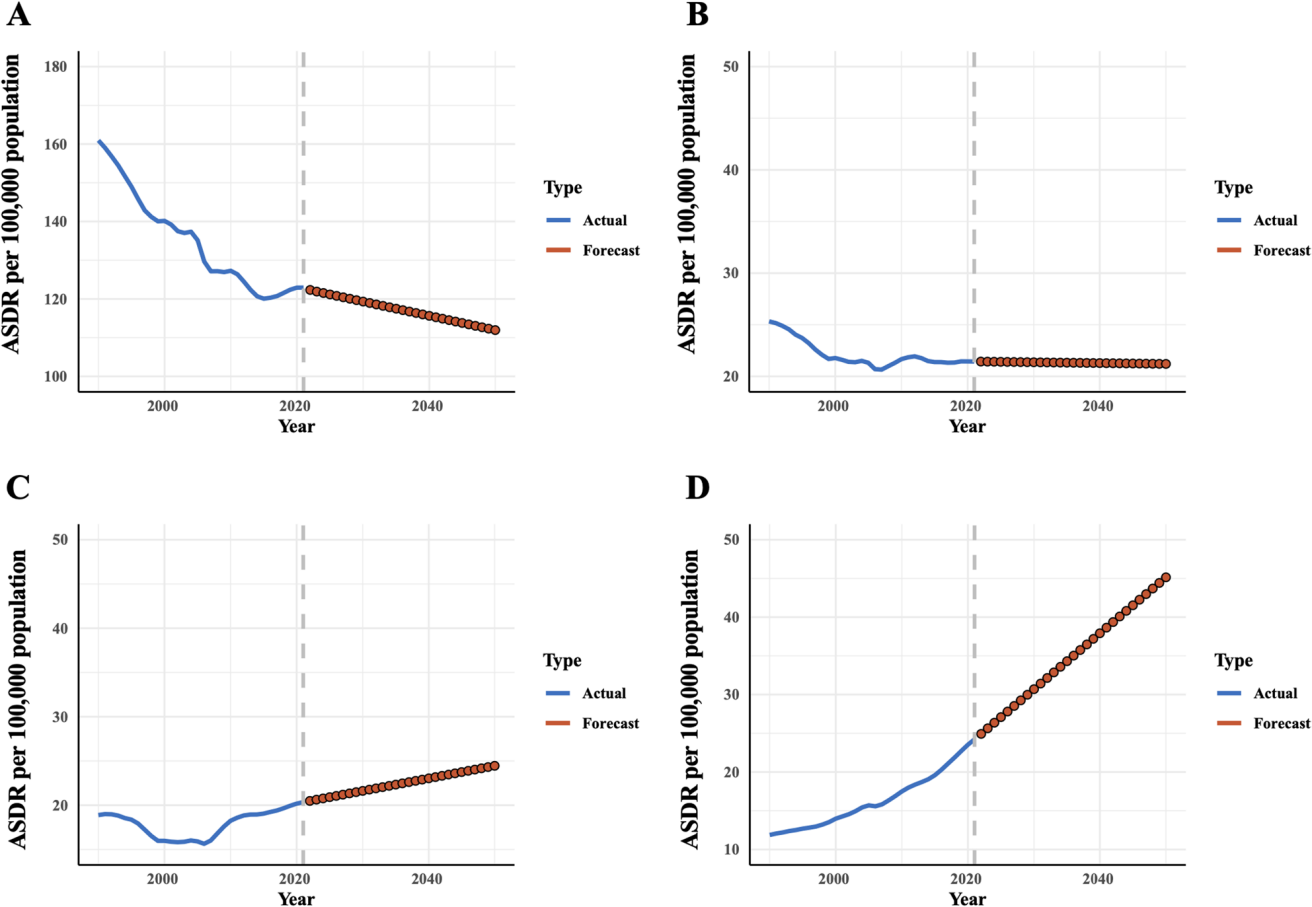
Future trends of DALY of CRC attributable to dietary risk (**A**), tobacco (**B**), alcohol (**C**), and high body mass index (**D**), respectively, were projected from 2022 to 2050 using ARIMA models. CRC: colorectal cancer; ARIMA: Autoregressive Integrated Moving Average. Solid blue line: observed GBD estimates (1990–2021); dark red circle: ARIMA-projected trends (2022–2050)

Future trends in the burden of colorectal cancer attributable to lifestyle habits as forecasted by the ARIMA model are presented in Fig. 4 and the Supplementary material. The data indicate a continued decline in the burden of CRC associated with dietary risks. Conversely, there is an anticipated in the burden of CRC attributable to high alcohol use and high BMI, with the latter exhibiting a particularly pronounced upward trajectory. In addition, the burden of colorectal cancer caused by smoking remains relatively stable.

## Discussion

Our original hypothesis was partially confirmed by our study, while the other part went in the opposite direction as we expected. Our study demonstrated that some lifestyle changes, such as diet habits, are indeed shifting towards healthier behaviors in China, and the disease burden of CRC attributable to this trend is also showing a decreasing trend, and our projections indicate that it will continue to decrease in the future. At the same time, however, the disease burden of CRC attributed to obesity is alarming, showing an obvious upward trend. In addition, the ARIMA prediction model indicated that the burden of disease attributable to smoking and obesity would continue to increase in the future, which should not be ignored. Dietary practices are significant risk factors for colorectal cancer and should not be overlooked due to their frequent occurrence in daily life. A low-fiber diet is associated with an elevated risk of colorectal cancer through several mechanisms [16]. Inadequate fiber intake may decrease insulin activity, thereby impairing glycemic control, and reduce the production of short-chain fatty acids, which possess anti-proliferative effects on colon cells [19]. Secondly, low-fiber diet can also prolong the transit time of intestinal contents in the intestine, thereby increasing the absorption of carcinogens [20]. Additionally, low fiber intake is often associated with low whole grain consumption, both of which are linked to increased CRC risk. Whole grains are rich sources of dietary fiber, vitamins, minerals, and phytochemicals, which are often significantly diminished during the processing of refined grains, thereby potentially promoting CRC [21, 22]. An intriguing finding of this study is the inverse relationship between low grain intake and low fiber intake observed in the Age-period-cohort analysis. We hypothesize that this trend may be influenced by the traditional Chinese diet, which frequently includes refined white rice and white flour products [23], which is caused by the long-standing preference for taste and appearance of food. Moreover, calcium ions are crucial in modulating the proliferation and differentiation of colonic cells. Inadequate calcium intake may disrupt these processes, potentially facilitating tumor development [24, 25]. Beyond calcium, milk contains vitamin D, conjugated linoleic acid, antioxidant lactoferrin, immunoglobulins, and other nutrients that have demonstrated anti-tumor properties, thereby contributing to a reduction in the disease burden of CRC [26, 27]. However, the prevalence of lactose intolerance within the Chinese population limits milk consumption, resulting in a negative correlation in the age-period-cohort analysis of period cohorts with low dietary intake of milk [28]. In addition, according to the assessment of the International Agency for Research on Cancer (IARC), processed meat has been classified as carcinogenic to humans (grade 1), whereas red meat is categorized as probably carcinogenic to humans (grade 2 A) [29]. The potential mechanisms for this classification include the formation of carcinogens, such as heterocyclic amines and polycyclic aromatic hydrocarbons, during the cooking of meat at high temperatures [30].

While our analysis shows declining age-standardized DALY rates attributable to low fiber and low calcium intake in China from 1990 to 2021, this should not be simplistically interpreted as evidence of “healthier” dietary behaviors. The exposure estimates underlying these GBD risk-outcome pairs are modeled constructs that integrate sparse survey data with covariates (e.g., education, urbanization), and their temporal changes may reflect methodological updates or data availability rather than true population-level shifts in consumption. Indeed, national nutrition surveys tell a more nuanced story: the China Nutrition and Health Surveillance (CNHS) reports persistent low intake of whole grains—far below the recommended 50–150 g/day—while processed meat consumption has risen steadily, particularly in urban areas. (https://chns.cpc.unc.edu/) Concurrently, increased fruit and vegetable intake may partly explain the decline in low-fiber burden, even as whole-grain consumption remains suboptimal. Therefore, the observed burden reductions likely reflect complex dietary transitions rather than uniform improvement. The inverse trend between low whole grain intake (increasing burden) and low dietary fiber intake (decreasing burden) warrants further investigation. This apparent contradiction can be explained by recognizing that dietary fiber in the Chinese diet derives not only from whole grains, but also potentially from vegetables, fruits, pulses, and tubers. We hypothesize that the transition towards a “modern” diet often carries dual effects: yielding benefits (such as increased fruit and vegetable intake) while simultaneously causing losses (such as the displacement of coarse grains). Consequently, an overall increase in dietary fiber intake does not necessarily equate to heightened whole grain consumption—a distinction crucial for formulating targeted public health interventions.

Smoking and high alcohol use have historically posed significant threats to human health [31, 32]. Empirical studies have shown that smokers have a substantially increased risk of developing CRC compared to non-smokers [33]. The underlying mechanism involves including DNA mutation or methylation, which subsequently affects oncogenes or proto-oncogenes, in addition to impairing the immune system by reducing the infiltration of T-cells and tumor-associated macrophages in the tumor, resulting in immune escape of tumor cells [34]. Alcohol is attributable to CRC through its metabolites, including DNA damage, interfering with the absorption of vitamin B and folate, and disruption of DNA repair mechanism [35]. According to Joinpoint regression analysis, while the disease burden associated with smoking showed an overall decline from 1990 to 2021, the deceleration began to slow down from 2007 and gradually showed an increasing trend.

Furthermore, China is currently facing an obesity and overweight epidemic, driven by nutritional transitions and lifestyle modifications (e.g., the rising proportion of ultra-processed foods in the Chinese diet and reduced physical activity), which is consistent with the global obesity problem [36–38]. Therefore, the burden of CRC associated with high BMI needs to be emphasized in China.

In conclusion, while the burden of colorectal cancer attributable to dietary risk factors has been significantly mitigated and is anticipated to further decline due to alterations in dietary patterns stemming from China’s rapid economic development, it is imperative to acknowledge that the burden associated with colorectal cancer remains substantial. Based on these findings, it is necessary to recalibrate public health priorities for CRC prevention in China. Whilst continuing to promote healthy dietary patterns remains important, urgent action is required to address the growing threat posed by high BMI and processed meat consumption. This necessitates the adoption of a comprehensive strategy.

Lastly, it cannot be ignored that there are still limitations in this study. First, all exposure and burden estimates are derived from the Global Burden of Disease (GBD) study, which relies on modeled imputations rather than direct nationwide measurements for many risk factors—particularly dietary components such as milk consumption, which may be inaccurately captured in Chinese contexts due to limited survey granularity or cultural reporting biases. Furthermore, although our analysis projects a sustained downward trend in disease burden through 2050, this projection is inherently limited by the statistical nature of the ARIMA model and the modeled origin of GBD inputs. These long-term forecasts should be viewed as indicative of potential trajectories under current trends, not as certain outcomes. Future research incorporating scenario-based modeling or hybrid approaches that integrate policy variables may provide more robust insights into plausible future burdens.

## Supplementary Information

Supplementary Material 1

## Data Availability

Data for this study can be obtained from the Global Health Data Exchange website (https://ghdx.healthdata.org/gbd-2021).
